# Polyphenols from persimmon fruit attenuate acetaldehyde-induced DNA double-strand breaks by scavenging acetaldehyde

**DOI:** 10.1038/s41598-022-14374-9

**Published:** 2022-06-18

**Authors:** Kenichiro Matsuzaki, Kenji Kumatoriya, Mizuki Tando, Takashi Kometani, Miki Shinohara

**Affiliations:** 1 Department of Advanced Bioscience, Graduate School of Agriculture, Kindai University, 3327-204 Nakamachi, Nara City, Nara 631-8505 Japan; 2 Agricultural Technology and Innovation Research Institute, Kindai University, 3327-204 Nakamachi, Nara City, Nara 631-8505 Japan; 3 Department of Food Science and Nutrition, Faculty of Agriculture, Kindai University, 3327-204 Nakamachi, Nara City, Nara 631-8505 Japan; 4 Pharma Foods International, Co., Ltd., 1-49 Goryo-Ohara, Nishikyo-ku, Kyoto, 615-8245 Japan

**Keywords:** DNA damage and repair, DNA adducts, DNA damage response, Double-strand DNA breaks, Homologous recombination, Non-homologous-end joining

## Abstract

Acetaldehyde, a metabolic product of ethanol, induces DNA damage and genome instability. Accumulation of acetaldehyde due to alcohol consumption or aldehyde dehydrogenase (*ALDH2*) deficiency increases the risks of various types of cancers, including esophageal cancer. Although acetaldehyde chemically induces DNA adducts, the repair process of the lesions remains unclear. To investigate the mechanism of repair of acetaldehyde-induced DNA damage, we determined the repair pathway using siRNA knockdown and immunofluorescence assays of repair factors. Herein, we report that acetaldehyde induces DNA double-strand breaks (DSBs) in human U2OS cells and that both DSB repair pathways, non-homologous end-joining (NHEJ) and homology-directed repair (HDR), are required for the repair of acetaldehyde-induced DNA damage. Our findings suggest that acetaldehyde-induced DNA adducts are converted into DSBs and repaired via NHEJ or HDR in human cells. To reduce the risk of acetaldehyde-associated carcinogenesis, we investigated potential strategies of reducing acetaldehyde-induced DNA damage. We report that polyphenols extracted from persimmon fruits and epigallocatechin, a major component of persimmon polyphenols, attenuate acetaldehyde-induced DNA damage without affecting the repair kinetics. The data suggest that persimmon polyphenols suppress DSB formation by scavenging acetaldehyde. Persimmon polyphenols can potentially inhibit carcinogenesis following alcohol consumption.

## Introduction

Maintenance of genome stability is crucial for cell proliferation. However, genomic DNA is exposed to various types of genotoxic stresses, which induce mutations and genome instability, if the DNA damage is not repaired or is misrepaired. In addition to environmental genotoxic stress, various endogenous metabolic products can induce DNA damage (e.g. reactive oxygen species, *S*-adenosyl methionine, and formaldehyde)^1^. It has been reported that acetaldehyde, a metabolic product of ethanol, induces DNA damage^2,3^. Acetaldehyde is produced from ethanol by alcohol dehydrogenase (ADH1B) and metabolised into acetic acid by aldehyde dehydrogenase (ALDH2)^4^. In *Aldh2*-deficient mice, accumulation of acetaldehyde causes DNA damage and promotes tumourigenesis, which is exacerbated by ethanol consumption^5^. Similarly, in humans, alcohol consumption and *ALDH2*-deficiency are strongly associated with the risk of various types of cancers, such as esophageal cancer^3,6–11^. Ethanol consumption also results in several physiological responses, including facial flushing, nausea, headaches, and increased heart rate^9,12^. Mutations in genes responsible for Fanconi anemia (FA) exacerbate *ALDH2*-deficient phenotypes^5^. FA genes are necessary for the repair of DNA interstrand cross-links (ICLs)^13^. During ICL repair, when the DNA replication machinery encounters ICLs, the FA core complex recognises the ICLs and ubiquitinates Fanconi anemia complementation group D2 (FANCD2)^13,14^. In previous studies, incisions were made at the ICL sites using XPF-ERCC1^13,14^. DNA double-strand breaks (DSBs) generated by incisions have been reported to be repaired via homologous recombination (HR).

Formaldehyde, which has a chemical structure similar to that of acetaldehyde, forms covalent bonds between DNA and proteins (DNA–protein cross-links; DPCs)^15^. Formaldehyde-induced DPCs are recognised as DNA damage in vivo and are repaired via the DPC repair pathway^15^. Similarly, acetaldehyde chemically induces ICLs and DPCs^2,3^. However, the types of genomic DNA damage induced by acetaldehyde and the resultant cellular responses are controversial. In terms of DPCs, it is unclear whether acetaldehyde induces DPCs in vivo and whether the DPC repair pathway is involved in the maintenance of genome stability after acetaldehyde treatment. In terms of ICLs, acetaldehyde chemically induces ICLs, and ICLs on plasmid DNA are repaired by several replication-dependent pathways in *Xenopus laevis* egg extracts^16^. However, it is unclear whether acetaldehyde induces ICLs in the human genome and whether such repair pathways are conserved in humans. Based on the accumulation of DNA damage in *Fancd2*- and *Aldh2*-double mutant mice^5^, it has been suggested that the FA/HR pathway is involved in the repair of acetaldehyde-induced DNA damage. In the FA/HR pathway, canonical ICLs induced by anticancer drugs, such as cisplatin and mitomycin C, are converted to DNA DSBs^13,14^. However, it is unclear whether acetaldehyde induces DSBs in a similar manner in human cells.

DSBs are the most severe forms of DNA damage and repaired via homology-directed repair (HDR) or non-homologous end-joining (NHEJ)^17^. DSBs are induced by ionising radiation or anticancer drugs, including PARP inhibitors, camptothecin, and etoposide. In the HR pathway, the most common pathway of HDR, DSB ends are resected by nucleases to produce 3′-overhanging single-stranded DNA (ssDNA)^18^. The generated ssDNA is bound by RAD51, which catalyses the homology search and strand invasion steps of HR by forming nucleoprotein filaments on ssDNA^19^. BRCA1 and BRCA2, whose mutations predispose individuals to breast and ovarian cancers, facilitate resection and RAD51 assembly on ssDNA, respectively. In the NHEJ pathway, DSBs are recognised and protected from DSB end resection^20,21^. The DSB ends are re-ligated using the DNA ligase IV complex^21^. Both pathways are regulated by the cell cycle. HR requires sister chromatids as homologous templates and is restricted to the S and G2 phases of the cell cycle. In contrast, NHEJ mainly functions in G1 phase. BRCA1 and BRCA2 are suggested to be involved in the repair of acetaldehyde-induced DNA damage^22^. To enhance our understanding of the risks of acetaldehyde exposure, it is important to determine the cellular responses. In addition, it is unclear whether HR and NHEJ participate in the repair of acetaldehyde-induced DNA in vivo.

In contrast to its DNA-damaging potential, acetaldehyde is useful in food processing. Acetaldehyde is used to remove astringency in persimmon fruit, *Diospyros*^23^. Persimmon polyphenols, known as tannins, are responsible for the astringent characteristics of persimmon fruit^23^. Persimmon polyphenols are composed of catechins, including epicatechin (EC), epicatechin gallate (ECg), epigallocatechin (EGC), and epigallocatechin gallate (EGCg)^23,24^. Because acetaldehyde efficiently removes astringency by reacting with persimmon polyphenols to produce non-astringent polyphenol aggregates^23,25^, the structure of persimmon polyphenols could be preferable for the reaction with acetaldehyde. Although the chemical reactions between acetaldehyde and polyphenols are well-documented^26–28^, the effect of persimmon polyphenols on the DNA-damaging potential of acetaldehyde remains unclear.

In the present study, we investigated the underlying mechanisms of repair of acetaldehyde-induced DNA damage in human cells. We report that acetaldehyde induces DSBs, which are repaired by HR and NHEJ. Our findings suggest that acetaldehyde-induced DNA damage is recognised as DSBs and repaired in a manner similar to canonical DSBs in human cells. Since persimmon polyphenols are not toxic to humans and use acetaldehyde to generate bridges between molecules, they are candidates for the entrapment of acetaldehyde and its detoxification. Therefore, we focused on the potential application of persimmon polyphenols to minimise the risk of acetaldehyde-associated carcinogenesis. Using immunofluorescence assay, we examined the effect of persimmon polyphenols on acetaldehyde-induced DNA damage. We showed that polyphenols from persimmon fruit and EGC attenuated acetaldehyde-induced DNA damage. Persimmon polyphenols did not affect the repair kinetics of acetaldehyde-induced DNA damage. Therefore, we propose that persimmon polyphenols and EGC detoxify acetaldehyde by scavenging it. Our findings could be applied to reduce DNA damage and cancer risk associated with alcohol consumption.

## Results

### DNA damage response pathway is activated by acetaldehyde treatment

**Figure 1:**
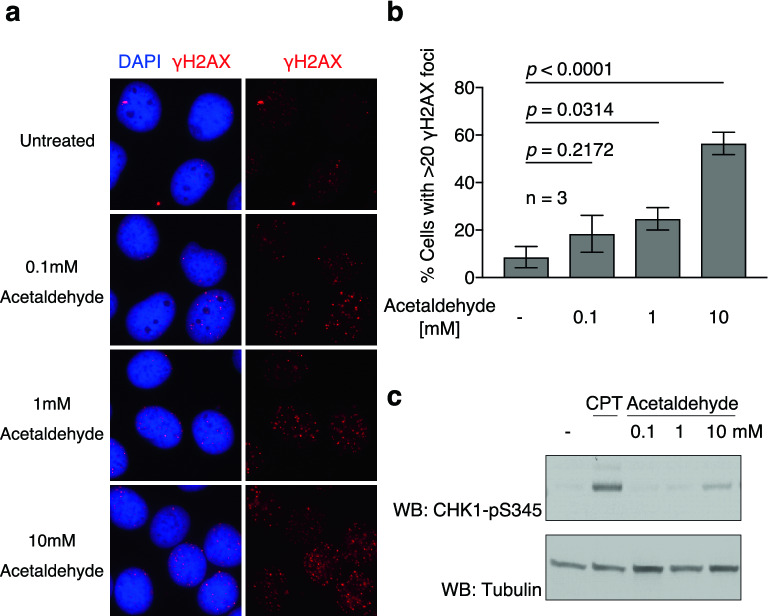
Exposure to acetaldehyde activates DNA damage response pathway. (**a**) Representative images of γH2AX foci without (untreated) or with the indicated concentrations of acetaldehyde in U2OS cells. (**b**) Frequency of γH2AX-positive cells untreated (−) or treated with the indicated concentrations of acetaldehyde. More than 100 cells were counted at each concentration. Statistical significance was determined using an unpaired *t* test. Error bars indicate standard deviation (n = 3). (**c**) CHK1-pS345 Western blot of whole cell extracts from HCT116 cells treated with the indicated concentrations of acetaldehyde or 100 nM camptothecin (CPT). Tubulin western blotting was used as a loading control.

Previous studies have reported that acetaldehyde can induce DNA damage^2,3^. To determine the optimal conditions for detecting acetaldehyde-induced DNA damage, γH2AX focus formation and CHK1 phosphorylation at serine 345 (CHK1-pS345) were assessed as key events in DNA damage responses. CHK1 is phosphorylated at Ser 345 by the DNA damage response ATR kinase after DSB formation or the stalling replication fork, and is thus known as potential marker for monitoring the level of DNA damage. Asynchronous U2OS cells were treated with the indicated concentrations of acetaldehyde and then analysed for γH2AX focus formation (Fig. 1a). γH2AX foci were observed even in untreated cells (8.6%), resulting from spontaneous DNA damage or replication stress. The frequency of γH2AX-positive cells was not significantly altered after 0.1 mM acetaldehyde treatment (18.4%; Fig. 1a, b). A slight but statistically significant increase in the frequency of γH2AX-positive cells was observed when cells were treated with 1 mM acetaldehyde (24.8%). A marked increase in the frequency of γH2AX-positive cells was observed when cells were treated with 10 mM acetaldehyde (56.5%) compared to that in untreated cells (Fig. 1a, b). Western blotting revealed that, consistent with the increased γH2AX focus formation, CHK1 phosphorylation signals were detected after 10 mM acetaldehyde treatment in HCT116 cells (Fig. 1c). Based on the increased γH2AX focus formation and CHK1 phosphorylation, we decided to use 10 mM acetaldehyde for subsequent experiments.

### DPC formation by acetaldehyde treatment is less efficient than that by formaldehyde

**Figure 2:**
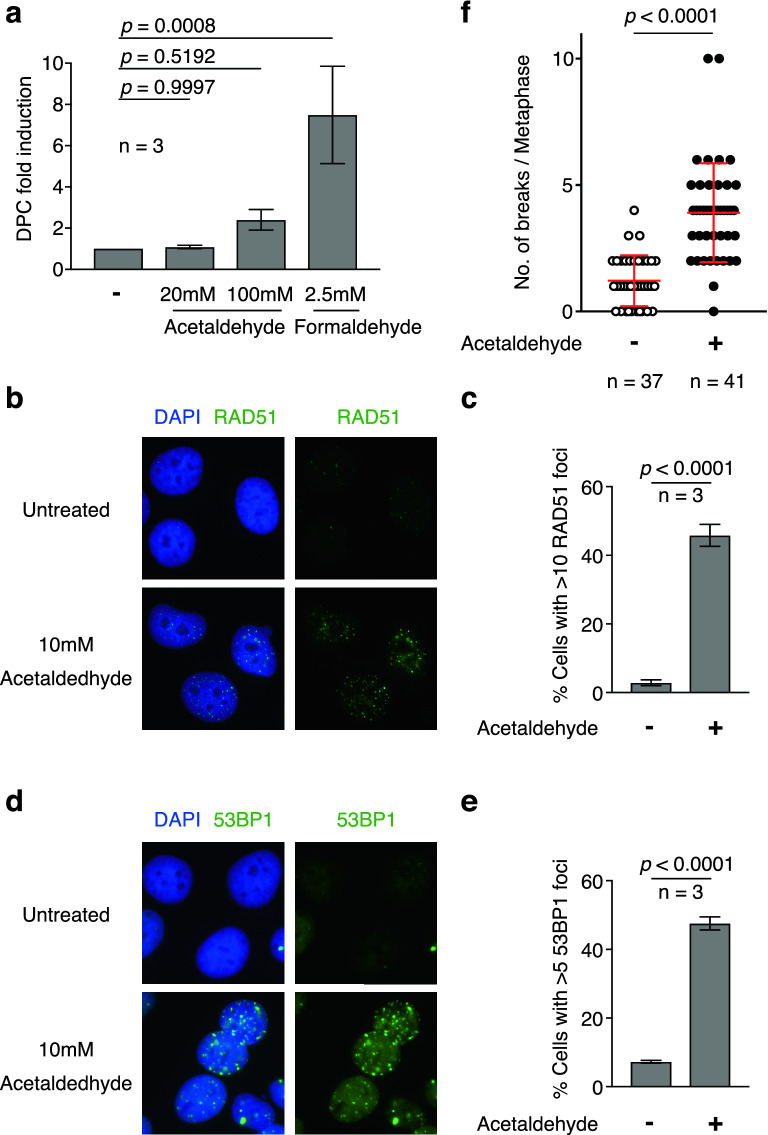
Acetaldehyde induces DNA double-strand breaks (DSBs). (**a**) Quantification of DNA–protein-cross links (DPCs) derived from U2OS cells treated with the indicated concentrations of acetaldehydes or formaldehyde. DPC amount was analysed using the ARK assay. The DPC-fold induction was calculated by normalising the amount of DPC in each sample to that of the untreated samples. Statistical significance was determined using an unpaired *t *test. Error bars indicate standard deviation (n = 3). (**b**) Representative image of RAD51 focus-positive U2OS cells with (10 mM acetaldehyde) or without (untreated) acetaldehyde. (**c**) Frequency of RAD51-positive cells with (+) and without (−) acetaldehyde treatment. More than 100 cells were counted for each condition. Statistical significance was determined using unpaired *t* test. Error bars indicate standard deviation (n = 3). (**d**) Representative image of 53BP1 focus-positive U2OS cells with (10 mM acetaldehyde) or without (untreated) acetaldehyde. (**e**) Frequency of 53BP1-positive cells with (+) and without (−) acetaldehyde treatment. More than 100 cells were counted for each condition. Statistical significance was determined using unpaired *t* test. Error bars indicate standard deviation (n = 3). (**f**) Distribution of the number of chromatid/chromosome breaks in metaphase-arrested HCT116 cells with (+) or without ( −) 10 mM acetaldehyde. More than 35 metaphase samples were analysed for each condition. Data are presented as the mean ± standard deviation. Statistical significance was determined using the Mann–Whitney *U* test.

To investigate the repair mechanisms of acetaldehyde-induced DNA damage, we first determined the types of DNA damage induced by acetaldehyde in vivo. Based on the similarity of its chemical structure to that of formaldehyde, acetaldehyde is believed to induce DPCs. A previous study suggested that acetaldehyde induces DPCs; however, there is still no direct evidence for the formation of DPCs by acetaldehyde^29^. Recently, a sensitive DPC detection assay (ARK assay) was developed^30^. ARK assays were used to assess the effects of acetaldehyde on DPC formation in vivo. As an experimental control, it was confirmed that 2.5 mM formaldehyde resulted in an eightfold increase in DPC formation compared to the untreated control, as previously reported^30^ (Fig. 2a). Subsequently, the relative amount of DPC after treatment with 20 mM acetaldehyde was indistinguishable from that in the untreated U2OS cells (Fig. 2a). Furthermore, treatment with 100 mM acetaldehyde increased DPC formation significantly (2.4-fold), although the effect was clearly less than that after treatment with 2.5 mM formaldehyde (Fig. 2a). To verify the effect of acetaldehyde on DPC formation, the amount of DPC was determined using rapid DNA adduct recovery (RADAR) assays, another assay for DPC recovery^31^. In the RADAR assay, proteins covalently bound to genomic DNA are recovered from genomic DNA prepared under denaturing conditions. The amount of DPC was determined by measuring the quantity of the DPC-associated protein moieties. Similar to the results of the ARK assay, DPC formation did not increase following acetaldehyde treatment in U2OS cells (Fig. S1). The data suggest that acetaldehyde induces DPCs less efficiently than formaldehyde. Considering the increased γH2AX focus formation and CHK1 phosphorylation in the presence of 10 mM acetaldehyde, such observations suggested that acetaldehyde induces little or no DPCs in human cells.

### *Acetaldehyde induces DSBs *in vivo

A previous study reported that human BRCA1/2-deficient cells are hypersensitive to acetaldehyde, suggesting that HR factors are involved in the repair of acetaldehyde-induced DNA damage^22^. To examine the involvement of HR in the repair of such damage, we analysed the focus formation of RAD51, which marks sites of HDR, in U2OS cells. The frequency of RAD51 focus-positive cells was significantly increased after acetaldehyde treatment (45.8%) (Fig. 2b, c). RAD51 accumulates at stalled replication forks and DNA DSBs^19,32^, suggesting that acetaldehyde leads to replication fork stalling or DSB formation. Previous reports have demonstrated that γH2AX focus formation increases after acetaldehyde treatment^33^. γH2AX focus formation is caused by DSB formation or replication fork stalling without breaks (e.g. DNA adducts, DNA polymerase inhibitor, depletion of the dNTP pool)^34,35^. It is challenging to distinguish whether acetaldehyde induces DSBs or stalling of replication forks based on focus formation of RAD51 and γH2AX. To distinguish between the two possibilities, we analysed the focus formation of 53BP1, which is a molecular marker of DSBs, and functions in the NHEJ pathway. An increased frequency of 53BP1 focus-positive cells was observed after acetaldehyde treatment (47.6%) (Fig. 2d, e). The observations suggest that acetaldehyde induces DSBs in the genomic DNA in vivo. To confirm whether acetaldehyde induced DSBs, the number of DNA breaks on metaphase chromosomes was measured in HCT116 cells (Fig. 2f). DNA breaks in metaphase chromosomes were significantly increased after acetaldehyde treatment compared to those in untreated cells (3.9% in acetaldehyde-treated cells and 1.2% in untreated cells) (Fig. 2f and S2a). Consistent with a previous report^22,33^, our observations indicate that acetaldehyde can induce DSBs in vivo and suggest that HR and NHEJ are involved in the repair of acetaldehyde-induced DSBs. Considering acetaldehyde is a highly reactive molecule, our observations prompted us to test whether acetaldehyde chemically cleaves the DNA directly. To this end, we incubated linearised plasmids with acetaldehyde and analysed their fragmentation. After 3 h of incubation at 37 °C, no fragmented DNA was detected (Fig. S2b). Therefore, acetaldehyde did not directly induce DSBs.

### HR and NHEJ are involved in the repair of acetaldehyde-induced DSBs

**Figure 3:**
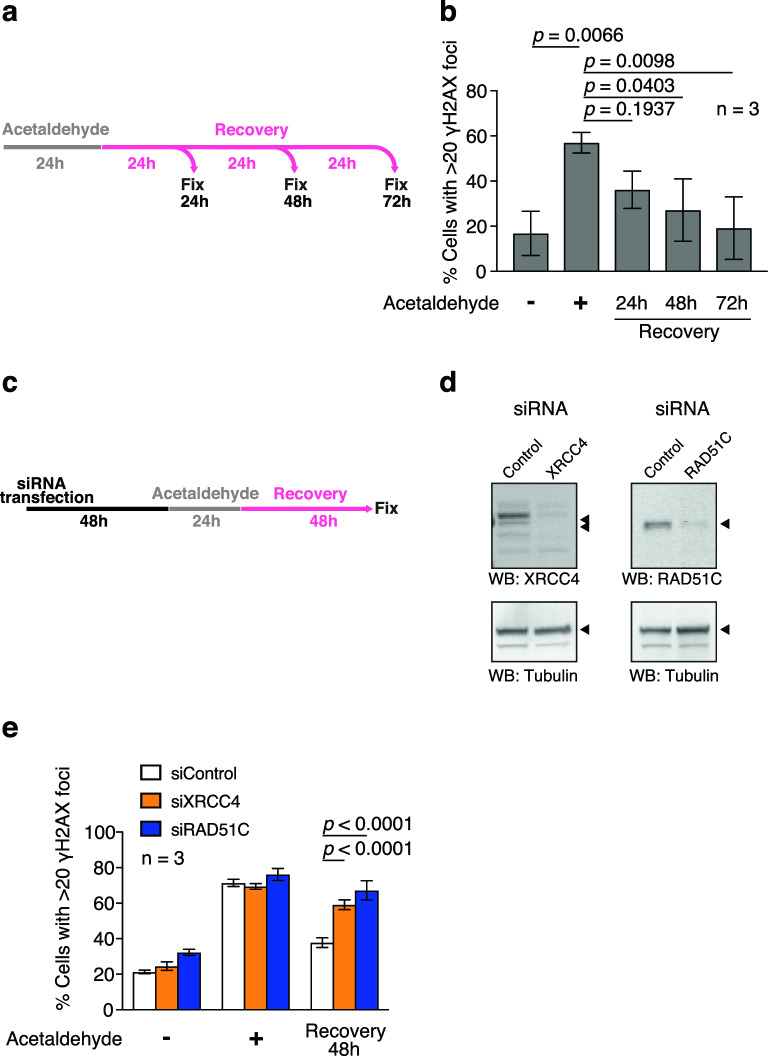
Homologous recombination and non-homologous end-joining are required for repair of acetaldehyde-induced DNA damage. (**a**) Schematic of the DNA repair kinetics experiment. U2OS cells were treated with 10 mM acetaldehyde for 24 h (grey). The cells were washed and incubated in an acetaldehyde-free culture medium (pink). At the indicated time points, cells were fixed and stained with an anti-γH2AX antibody. (**b**) Frequency of γH2AX-positive cells at the indicated time points after acetaldehyde treatment. More than 100 cells were counted in each sample. Statistical significance was determined using unpaired *t *test. Error bars indicate standard deviation (n = 3). (**c**) Schematic of repair kinetics experiments in RAD51C- or XRCC4-depleted cells. U2OS cells were transfected with siRNA against RAD51C or XRCC4. Forty-eight hours after transfection, the cells were treated with 10 mM acetaldehyde for 24 h. Subsequently, the cells were washed and incubated in acetaldehyde-free medium. Forty-eight hours after release, cells were fixed and stained with an anti-γH2AX antibody. (**d**) Western blotting of whole-cell extracts of XRCC4- or RAD51C-depleted cells to validate the expression of each protein. Tubulin was used as a loading control. (**e**) Frequencies of γH2AX-positive cells in RAD51C- or XRCC4-depleted U2OS cells. More than 100 cells were counted for each condition. Statistical significance was determined using unpaired *t *test. Error bars indicate standard deviation (n = 3).

DSBs are mainly repaired using either HR or NHEJ. A few reports have shown that deficiencies in FA/HR factors cause DNA damage and reduce survival^5,22^. However, whether such factors are required for the repair of acetaldehyde-induced DNA damage and repair kinetics is poorly understood. Because RAD51 and 53BP1 accumulate in acetaldehyde-induced DSBs, we examined whether HR and NHEJ are required for the repair of acetaldehyde-induced DSBs. To this end, we first analysed the DNA repair kinetics of acetaldehyde-induced DSBs in U2OS cells by monitoring the γH2AX foci as an indicator of DNA damage. After treatment with acetaldehyde for 24 h, 57.0% of cells were detected as being γH2AX-positive. The cells were then washed and released into acetaldehyde-free medium to monitor their recovery from acetaldehyde-induced DSBs. At 24, 48, and 72 h after release, γH2AX focus formation was assessed (Fig. 3a). At 24 h, a reduction in γH2AX-positive cells was observed, although the difference was not statistically significant (Fig. 3b). At 48 and 72 h, the number of γH2AX-positive cells decreased to background levels (27.2% and 19.2%, respectively), indicating that acetaldehyde-induced DSBs were repaired within 72 h of release. To test whether HR and/or NHEJ are required for the repair of acetaldehyde-induced DSBs, we depleted RAD51C or XRCC4, key components of HR and NHEJ, respectively, from U2OS cells using specific siRNAs (Fig. 3c). The repair of acetaldehyde-induced DSBs was assessed by monitoring the formation of γH2AX foci (Fig. 3d). Forty-eight hours after release from acetaldehyde treatment, much higher levels of γH2AX-positive cells were observed in RAD51C- and XRCC4-depleted cells, compared to those in the control cells (67.2% in RAD51C-depleted cells, 59.1% in XRCC4-depleted cells, 37.8% in control cells) (Fig. 3e). The data indicate that the repair of acetaldehyde-induced DSBs was delayed in both HR- and NHEJ-deficient cells. Therefore, HR and NHEJ are required for the repair of acetaldehyde-induced DSBs.

### Persimmon fruit extract attenuates acetaldehyde-induced DSB formation

Acetaldehyde induces DNA damage and genome instability, which promotes carcinogenesis^3^. Indeed, acetaldehyde accumulation during alcohol consumption increases the risk of cancer in humans and mice^10,11^. Compounds that detoxify acetaldehyde may be useful for inhibiting carcinogenesis. To identify such detoxifying compounds, we focused on persimmon fruit. Traditionally, acetaldehyde, which is produced from ethanol, has been used to remove astringency in persimmon fruit^23^. During the removal process, acetaldehyde efficiently reacts with polyphenols from the fruit, leading to the formation of insoluble and non-astringent polyphenol aggregates through a non-enzymatic process^23,25^. Since insoluble complexes composed of acetaldehyde and polyphenols are unable to penetrate cell membranes, we considered the possibility that polyphenols from persimmon fruit might detoxify acetaldehyde.

**Figure 4:**
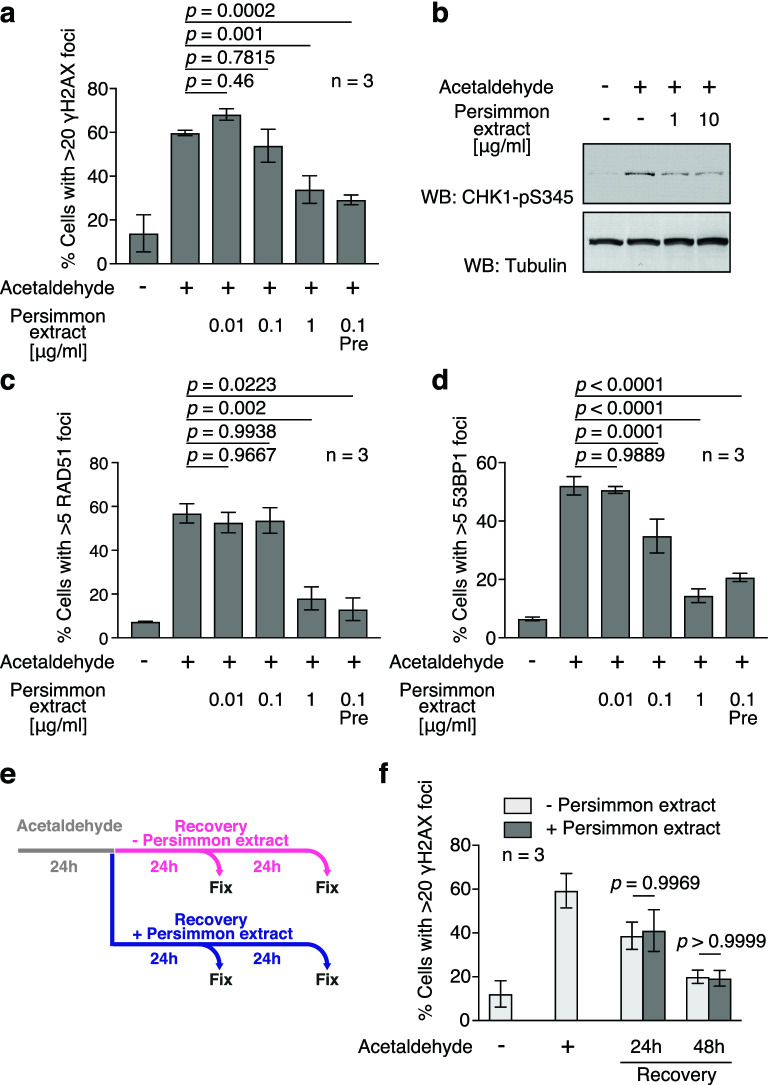
Persimmon fruit extract attenuates acetaldehyde-induced DNA damage. (**a**) Frequencies of γH2AX-positive cells treated with (+) or without (−) acetaldehyde and indicated concentrations of persimmon polyphenols. U2OS cells were incubated in medium containing 10 mM acetaldehyde and indicated concentrations of the persimmon polyphenol fraction for 24 h and subjected to immunofluorescence staining. Pre: Acetaldehyde was pre-incubated with persimmon polyphenol fraction for 1 h and added to the medium. For each condition, > 100 cells were counted. Statistical significance was determined using unpaired *t *test. Error bars indicate standard deviation (n = 3). (**b**) CHK1-pS345 western blot of whole cell extracts from HCT116 cells in the presence of acetaldehyde and indicated concentrations of the persimmon polyphenol fraction. (**c**) Frequency of RAD51-positive cells treated with acetaldehyde and persimmon polyphenols. U2OS cells were incubated in medium containing 10 mM acetaldehyde and indicated concentrations of the persimmon polyphenol fraction for 24 h and subjected to immunofluorescence staining. Pre: Acetaldehyde was pre-incubated with persimmon polyphenol fraction for 1 h and added to the medium. For each condition, > 100 cells were counted. Statistical significance was determined using unpaired *t *test. Error bars indicate standard deviation (n = 3). (**d**) Quantification of 53BP1-positive cells treated with acetaldehyde and persimmon polyphenols. U2OS cells were incubated in medium containing 10 mM acetaldehyde and indicated concentrations of the persimmon polyphenol fraction for 24 h and subjected to immunofluorescence staining. Pre: Acetaldehyde was pre-incubated with persimmon polyphenol fraction for 1 h and added to the medium. For each sample, > 100 cells were counted. Statistical significance was determined using unpaired *t *test. Error bars indicate standard deviation (n = 3). (**e**) Schematic of experiments showing DNA repair kinetics with persimmon polyphenols after acetaldehyde treatment. U2OS cells were treated for 24 h with acetaldehyde, then washed and incubated in the presence or absence of 1 µg/ml persimmon fruit extract. The cells were fixed and analysed by immunofluorescence staining at the indicated time points. (**f**) Quantification of γH2AX-positive cells at the indicated time points after acetaldehyde treatment. For each sample, > 100 cells were counted. Statistical significance was determined using unpaired *t *test. Error bars indicate standard deviation (n = 3).

To assess the effect of persimmon polyphenols on acetaldehyde-induced DNA damage, we simultaneously added acetaldehyde and the polyphenol fraction prepared from persimmon fruit to the media and monitored γH2AX focus formation (Fig. 4a). After acetaldehyde treatment in the absence of persimmon polyphenols, 60.7% of the cells were determined as being γH2AX focus-positive in U2OS cells. In the presence of 1 µg/ml and 0.1 µg/ml persimmon polyphenol fraction, the frequency of γH2AX focus-positive cells was reduced to 33.9% and 53.9%, respectively (Fig. 4a). To verify the effect of persimmon polyphenols on acetaldehyde-induced DNA damage, CHK1 phosphorylation and focus formation of RAD51 and 53BP1 were assessed. CHK1 phosphorylation was reduced by the addition of 1 µg/ml and 10 µg/ml persimmon polyphenol fractions to HCT116 cells (Fig. 4b). Consistent with the reduction in γH2AX focus formation, the frequency of 53BP1- and RAD51-focus positive cells was significantly decreased in the presence of the 1 µg/ml polyphenol fraction in U2OS cells (Fig. 4c, d). Intriguingly, although the simultaneous addition of acetaldehyde and the 0.1 µg/ml persimmon polyphenol fraction had minimal or no effect on γH2AX, RAD51, and 53BP1 focus formation, a significant decrease was observed when the same concentration of persimmon polyphenol fraction was pre-incubated with acetaldehyde (Fig. 4a, c, d). Such observations suggest that persimmon polyphenols attenuate the DNA damaging potential of acetaldehyde. Alternatively, persimmon polyphenols may facilitate repair of acetaldehyde-induced DNA damage. To exclude the latter possibility, we assessed their effect on repair by adding polyphenols after inducing DNA damage in U2OS cells (Fig. 4e). At 24 and 48 h after the removal of acetaldehyde with or without persimmon polyphenols, the frequency of γH2AX-positive cells was not altered by the addition of the persimmon polyphenol fraction (Fig. 4f). This observation suggests that persimmon polyphenols detoxify acetaldehyde by attenuating its DNA-damaging potential rather than by facilitating DNA repair, although we cannot exclude the possibility that polyphenols themselves affect cellular responses, such as gene expression or post-translational modifications.

### EGC is an acetaldehyde scavenger and attenuates acetaldehyde-induced DNA damage

**Figure 5:**
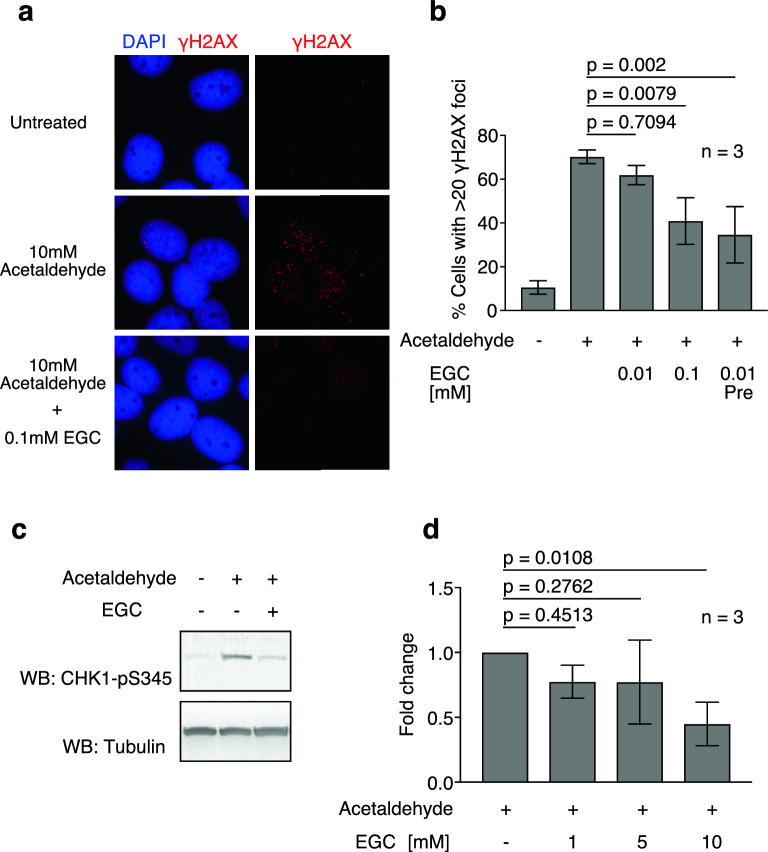
Epigallocatechin (EGC) attenuates acetaldehyde-induced DNA damage by scavenging acetaldehyde. (**a**) Representative images of γH2AX foci in U2OS cells treated with acetaldehyde and the indicated EGC concentrations. (**b**) Frequency of γH2AX-positive cells treated with acetaldehyde (+) and the indicated EGC concentrations. Pre: Acetaldehyde was pre-incubated with EGC for 1 h and added to the medium. More than 100 cells were counted for each condition. Statistical significance was determined using unpaired *t *test. Error bars indicate standard deviation (n = 3). (**c**) CHK1-pS345 western blot of whole-cell extracts from HCT116 cells in the presence of acetaldehyde and EGC. (**d**) Quantification of acetaldehyde after incubation with the indicated EGC concentrations. The acetaldehyde-fold change was calculated by normalising the acetaldehyde concentration in each sample to that in the absence of EGC. Statistical significance was determined using unpaired *t *test. Error bars indicate *the* standard deviation (n = 3).

Persimmon polyphenols (tannins) are complex and heterogeneous forms of polymerised polyphenols. The reduced DNA damage after treatment with the persimmon polyphenol fraction prompted us to test whether the components of persimmon polyphenols could detoxify acetaldehyde. A previous study has reported that persimmon polyphenols consist of epicatechin (EC), epicatechin gallate (ECg), epigallocatechin (EGC) and epigallocatechin gallate (EGCg)^24^. Among these, the major component is EGC^23,24^. Therefore, we examined the effects of EGC on acetaldehyde-induced DNA damage in U2OS cells. To this end, we monitored γH2AX focus formation in the presence or absence of EGC. In the presence of 0.1 mM EGC, the frequency of γH2AX focus-positive cells decreased to 40.9%, whereas 70.2% were observed after acetaldehyde treatment only (Fig. 5a, b). Similar to the persimmon polyphenol fraction, pre-incubation with acetaldehyde and 0.01 mM EGC efficiently attenuated acetaldehyde-induced DNA damage (Fig. 5a, b). Consistent with the reduced γH2AX focus formation, CHK1 phosphorylation was also decreased by EGC treatment in HCT116 cells (Fig. 5c). The observations suggest that persimmon polyphenols can detoxify acetaldehyde.

A previous study reported that acetaldehyde bridges catechins and produces catechin polymers, raising the possibility that catechins, including EGC, scavenge acetaldehyde^23,26^. In that case, acetaldehyde can be used for the polymerisation of catechins, and the concentration of acetaldehyde can be reduced. To examine this possibility, we measured acetaldehyde concentration after incubation with EGC using an acetaldehyde detection kit. Incubation with 1 mM, 5 mM, and 10 mM EGC decreased acetaldehyde present in the reaction by 23.5%, 23.8%, and 55.1%, respectively, relative to that in the absence of EGC (Fig. 5d). The observations suggest that EGC scavenges acetaldehyde and attenuates acetaldehyde-induced DNA damage.

## Discussion

**Figure 6:**
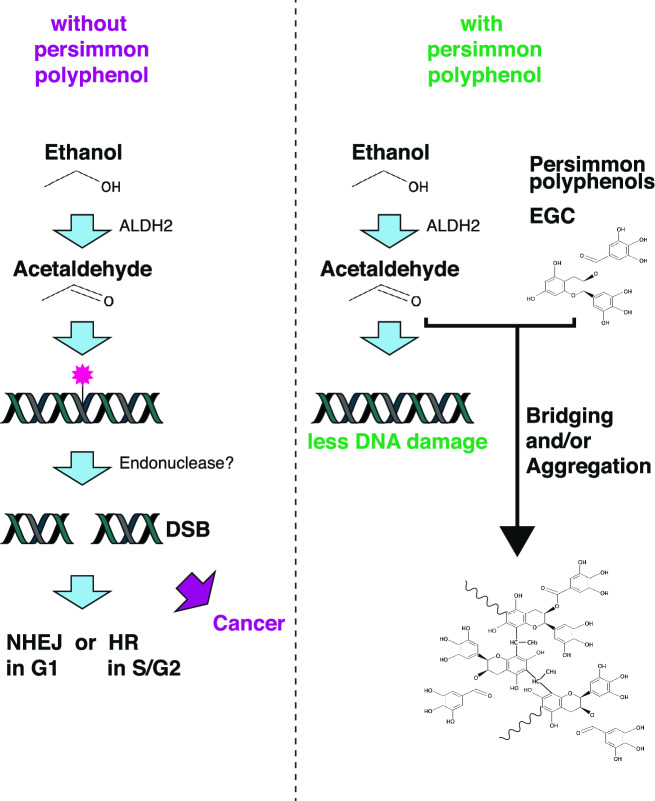
Model of acetaldehyde-induced DNA damage and maintenance of genome stability by persimmon polyphenols. (Left) In the absence of persimmon polyphenols, acetaldehyde produced from ethanol induces DNA adducts/interstrand cross-links (ICLs). In the S phase of the cell cycle, replication forks encounter acetaldehyde-induced DNA damage and are recognised by FA pathway components and DSBs are generated by nucleolytic incision. Double-strand breaks (DSBs) in S phase are repaired by homologous recombination (HR). In G1 phase, acetaldehyde-induced DNA damage might be recognised and cleaved by unidentified endonuclease to generate DSBs. DSBs in G1 phase are repaired by non-homologous end-joining (NHEJ). (Right) In the presence of persimmon polyphenols or EGC, acetaldehyde bridges polyphenols or is used for polyphenol aggregation. As a result, acetaldehyde is removed from the medium.

Several studies have reported that acetaldehyde induces DNA damage^2,3^. However, the type of DNA damage induced by acetaldehyde and its cellular responses remain largely unclear. Here, using immunofluorescence assay of DNA repair factors, we found that acetaldehyde induced DSBs, which are repaired by DSB repair pathways, HDR and NHEJ (Fig. 6). Our observations suggest that acetaldehyde-induced DNA damage is recognised as a DSB in vivo. In addition, because acetaldehyde is generated during the metabolic processing of ethanol, it is important to develop a method to reduce the DNA-damaging potential of acetaldehyde to inhibit carcinogenesis. Based on the chemical reaction between acetaldehyde and polyphenols, we focused on polyphenols and established that polyphenols from persimmon fruit detoxified acetaldehyde by reducing DNA damage. The result implies that the intake of persimmon fruit reduces the risk of acetaldehyde-associated carcinogenesis. Overall, we determined the repair pathway of acetaldehyde-induced DSBs and suggested the potential use of persimmon polyphenols in detoxification.

Previous studies have shown that acetaldehyde chemically modifies nucleotides, such as DPCs, ICLs, and DNA adducts^2,3,36^. However, the type of DNA damage induced by acetaldehyde in the cells remains controversial. Here, we found that DSBs are induced by acetaldehyde and recognised by the HDR machinery protein RAD51 and NHEJ protein 53BP1 (Fig. 2b-e). Previous studies have suggested that acetaldehyde induces DPCs^29^. Using sensitive DPC detection assays, we found that acetaldehyde induced little or no DPCs in genomic DNA (Fig. 2a, S1). Thus, it seems that DPCs are not a major cause of genome instability induced by acetaldehyde and that the DPC repair pathway is not necessary to maintain genome stability after acetaldehyde treatment. In terms of ICLs, it has been reported that acetaldehyde induces ICLs and that acetaldehyde-induced ICLs are repaired by the FA pathway or another replication fork convergence-dependent pathway in a *X. laevis* egg extract system^16^. In the general mechanism of FA pathway-dependent ICL repair, replication forks encounter ICLs induced by DNA crosslinkers, such as cisplatin, and XPF-ERCC1, a structure-specific endonuclease, is recruited to create nucleolytic incisions that generate DSBs^13,14^. Such DSBs are repaired by HDR^13,14^. In the present study, we were unable to provide direct evidence that acetaldehyde induces ICLs in the human genomic DNA. However, our finding that acetaldehyde treatment increased RAD51 focus formation and the requirement of RAD51C for repair implied that acetaldehyde-induced ICLs, which are converted to DSBs, are repaired in a manner similar to the repair of canonical ICLs during the S phase of the cell cycle. Such observations are consistent with the requirement of BRCA1/2 for survival after acetaldehyde treatment^22^. Furthermore, we showed the cytological kinetics of the repair (Fig. 3b). Recently, several replication-dependent pathways repair ICLs^16,37^. Although we demonstrated the kinetics of the repair, it is difficult to directly detect acetaldehyde-induced ICLs in vivo and investigate the relationships among these repair pathways in human cells. In future, techniques for detecting or visualising acetaldehyde-induced ICL/DSB should be developed.

In contrast to replication-dependent repair, NHEJ repairs DSBs in a replication-independent manner and primarily functions in the G1 phase of the cell cycle^21^. Our findings that acetaldehyde induced 53BP1 focus formation (Fig. 2d, e) and that XRCC4 is required for the repair of acetaldehyde-induced DSBs (Fig. 3e) imply that, in the G1 phase, DSBs are induced by acetaldehyde in a replication-independent manner and repaired by the NHEJ pathway. Unexpectedly, direct cleavage of DNA strands was not observed after the incubation of DNA molecules with acetaldehyde (Fig. S2b). Thus, acetaldehyde treatment indirectly cleaved DNA strands in vivo. Because acetaldehyde potentially gives rise to bulky adducts, such adducts can be directly recognised and cleaved by specific endonucleases during the repair process in G1 phase. Acetaldehyde forms guanine adducts, including covalent guanine dimers and ICLs^2,3,36^. Similar to pyrimidine dimers, covalent guanine dimers may be recognised and cleaved by the nucleotide excision repair pathway in a replication-independent manner. Alternatively, guanine-rich sequences may form four-stranded secondary structures, G-quadruplexes (G4), and acetaldehyde may stabilise G4 structures. A recent study showed that stabilised G4 structures are recognised in a transcription-dependent manner and converted to DSBs by topoisomerase 2 activity^38^, which implies that acetaldehyde-induced guanine adducts are recognised by a similar mechanism. Our findings suggest that acetaldehyde induces DSBs in replication-dependent and replication-independent manners. In contrast to the well-characterised replication-dependent DSB formation, little is known about the replication-independent pathways. Therefore, the replication-independent DSB formation pathway mechanism requires further investigation.

DSBs cause genome instability and promote carcinogenesis. Indeed, mutations involving HDR or NHEJ genes, such as *Brca1*, *Brca2*, *Mre11*, *Nbs1*, and *Lig4*, predispose patients to several types of cancers^19,39^. Previous studies have reported that alcohol intake is associated with an increased risk of several types of cancer^10,11^. Considering acetaldehyde is a metabolic intermediate of ethanol that induces DNA damage, acetaldehyde-induced DNA damage could be a cause of the observed increase in cancer risk due to alcohol consumption. It is important to detoxify acetaldehyde to inhibit alcohol-induced carcinogenesis provoked by alcohol consumption. Based on the reactivity of persimmon polyphenols with acetaldehyde (Fig. 6)^23,25^, we investigated whether persimmon polyphenols could detoxify acetaldehyde. Intriguingly, the persimmon polyphenol fraction and EGC, a major component of persimmon polyphenols, attenuated acetaldehyde-induced DNA damage (Figs. 4 and 5). The effects of the persimmon polyphenol fraction and EGC include (1) pre-incubation of persimmon polyphenol or EGC with acetaldehyde effectively reduced DNA damage; (2) incubation of acetaldehyde with EGC decreased acetaldehyde concentrations^26^; and (3) repair kinetics of acetaldehyde-induced DSBs were not altered by the addition of the persimmon polyphenol fraction. Therefore, persimmon polyphenols attenuate acetaldehyde-induced DNA damage by scavenging acetaldehyde rather than facilitating DNA repair (Fig. 6). Such data suggest that the detoxification of acetaldehyde by persimmon polyphenols may reduce the risk of cancer caused by alcohol consumption. Acetaldehyde forms bridges between catechins^23^. Therefore, similar reactions generate covalent bonds between the persimmon polyphenols. Since the structures of persimmon polyphenols are complex forms of polymerised polyphenols, persimmon polyphenols have numerous contact surfaces between molecules, which makes the reaction highly efficient.

Following alcohol consumption, more than 30% of East Asians and 8% of the world population exhibit an alcohol flushing response due to inherited deficiency of *ALDH2*, which catalyses the reaction from acetaldehyde to acetic acid^12^. In addition to the alcohol flushing response, *ALDH2* deficiency increases the risk of alcohol-related cancer^10,11^. Based on the reduction in acetaldehyde-induced DNA damage by persimmon polyphenols, we propose that persimmon intake can potentially minimise the flushing response and future risk of cancer caused by alcohol consumption. In the present study, the effects of persimmon polyphenols on tissues and the body after ethanol intake were not tested. Experiments using animals with or without *ALDH2* mutations are required to verify acetaldehyde detoxification by polyphenols in tissues and bodies.

## Methods

### Cell culture

All cell lines used in the present study were obtained from the American Type Culture Collection (ATCC). U2OS cells were used for immunofluorescence analysis because they express wild-type p53 and are suitable for use in analyses DNA damage responses and apoptosis. HCT116 cells were used for metaphase spread and western blotting analyses because they have a stable karyotype and lower level of spontaneous DNA damage. Both cell lines were immortalised. Cells were cultured in Dulbecco’s modified Eagle’s medium supplemented with 10% fetal bovine serum and 1% antibiotic–antimycotic mixed solution (Nacalai, 02892-54). All experiments were performed in accordance with institutional guidelines and regulations approved by the Kindai University Genetic Recombination Experiment and Biosafety Committee.

### Antibodies

Anti-γH2AX (Millipore 05-636), anti-RAD51 (Millipore ABE257), and anti-53BP1 (Novus Biologicals NB100-304) antibodies were used for the immunofluorescence analysis. Anti-CHK1-pS345 (Cell Signalling Technology 2348), anti-XRCC4^40^, anti-RAD51C (Novus Biologicals NB100-177), and anti-tubulin (Sigma, T4026) antibodies were used for the western blot analysis.

### Western blot

HCT116 and U2OS cells were resuspended in benzonase buffer (20 mM Tris–HCl (pH 7.5), 40 mM NaCl, 2 mM MgCl_2_, 0.5% NP-40, 50 U/ml benzonase, protease inhibitor cocktail, PhosSTOP (Roche, 4906845001), and incubated on ice for 10 min. NaCl (5 M) was then added to the samples at a final concentration of 450 mM. The samples were rotated at 4 °C for 30 min and then centrifuged at 13,000 rpm for 10 min. The supernatants were separated using 15% SuperSep Ace (Wako, 193-14991) and transferred onto PVDF membranes. The membranes were blocked with 5% skim milk and subsequently incubated with primary antibodies at 4 °C overnight. After overnight incubation, the membranes were incubated with AP-conjugated secondary antibodies and treated with a BCIP-NBT alkaline phosphatase solution (Nacalai, 03937-60) for detection. Images were cropped and processed using Photoshop 2020 (Adobe, USA).

### Immunofluorescence staining

U2OS cells cultured on coverslips were permeabilised with CSK buffer (10 mM PIPES pH 6.8, 100 mM NaCl, 300 mM sucrose, 3 mM MgCl_2_, 1 mM EDTA, 0.5% Triton-X100, protease inhibitor cocktail, PhosSTOP) for 5 min on ice. After permeabilisation, cells were fixed in 2% paraformaldehyde at room temperature (25 °C) for 15 min, then blocked in blocking buffer (PBS containing 10% goat serum, 5% BSA, 0.1% Triton X-100) at room temperature for 30 min. After blocking, the coverslips were incubated in blocking buffer containing the primary antibody at 4 °C overnight. Coverslips were washed in PBS containing 0.1% Triton X-100 three times and incubated with secondary antibodies at room temperature for 1 h. The cells were stained with DAPI and mounted using Vectashield (Vector Laboratories, H-1000).

### ARK assay

The ARK assays were performed as described previously^30^. Briefly, cells were collected by scraping and resuspended in M buffer (5.6 M GTC, 10 mM Tris–HCl [pH 6.8], 20 mM EDTA, 4% Triton X-100, 1% Sarkosyl, 1% DTT). The samples were sheared by passing through a 22 G needle. After adding an equal volume of ethanol, the samples were centrifuged at 13,000 rpm for 20 min. The precipitate was resuspended in 1% SDS/20 mM Tris–HCl (pH 7.5) and incubated at 42 °C for 6 min. After DNA shearing using 25 G needles, the DPCs were precipitated by adding 200 mM KCl/20 mM Tris–HCl (pH 7.5). The DPCs were precipitated by centrifugation and resuspended in 100 mM KCl and 20 mM Tris–HCl (pH 7.5). The samples were incubated at 55 °C for 10 min, and then at 4 °C for 6 min. DPCs were precipitated by centrifugation and washed with 100 mM KCl and 20 mM Tris–HCl (pH 7.5). Samples were resuspended in ProK buffer (100 mM KCl, 20 mM Tris–HCl (pH 7.5), 10 mM EDTA, 0.2 mg/ml proteinase K), and incubated at 55 °C for 45 min. After centrifugation, supernatants containing DPC-associated DNA were collected, and DNA concentrations were determined using a Qubit 1 × dsDNA HS assay kit (Invitrogen, Q33230). The relative values of DPC/input to untreated samples are presented as ‘DPC-fold induction’.

### RADAR assay

After acetaldehyde or formaldehyde treatment, cells were harvested by scraping and resuspended in 500 µL of RADAR lysis buffer (6 M GTC, 10 mM Tris–HCl [pH 6.8], 20 mM EDTA, 4% Triton X-100, 1% Sarkosyl, 1% DTT). An aliquot of cell lysate (20%) was saved as an ‘input’ sample. An equal volume of 100% ethanol was added to the remaining cell lysate. After incubation at − 20 °C for 5 min, the samples were centrifuged at 15,000 rpm for 15 min. The precipitates were washed once with wash buffer (20 mM Tris–HCl (pH 6.8), 150 mM NaCl, 50% EtOH) and twice with 70% EtOH. The precipitated DPCs were resuspended in 200 µL 8 mM NaOH. Protein concentrations in the DPC samples and inputs were determined using a Qubit Protein Assay Kit (Invitrogen, Q33211). The relative values of DPC/input to untreated samples are presented as ‘DPC-fold induction’.

### Metaphase spreads

HCT116 cells incubated in the presence or absence of 10 mM acetaldehyde for 48 h were treated with 0.2 µg/ml colcemid (Gibco, 15212012) for 90 min and collected by washing the plate with culture medium. After washing with PBS, 5 ml of 0.075 M KCl was added to the cell suspension, which was then incubated at 37 °C for 15 min. Five millilitres of fixative (MeOH:AcOH = 3:1) was added to the cell suspension and the solution centrifuged at 1000 rpm for 5 min. The cell precipitates were resuspended in fixative and incubated on ice for 15 min. The cells were collected by centrifugation and washed once with a fixative. Metaphase spreads were prepared by dropping the cell suspension onto wet glass slides and staining the slides using the Giemsa method. More than 35 metaphases were analysed for each condition. The total number of chromatids and chromosome breaks is presented as ‘No. of breaks/metaphase’.

### siRNA transfection

U2OS cells were transfected with siRNA using the RNAiMAX transfection reagent (Invitrogen, 13778100). Briefly, 4 µL of 50 µM duplex RNA and 10 µL of RNAiMAX were added to 500 µL Opti-MEM (Gibco, 31985062) and incubated at room temperature for 20 min. Opti-MEM containing siRNA and RNAiMAX was added to the medium. Acetaldehyde was added to the medium 48 h after transfection. The medium was replaced with an acetaldehyde-free medium 24 h after acetaldehyde treatment. Cells were fixed at the indicated time points and subjected to immunofluorescence analysis.

siControl; UAGCGACUAAACACAUCAA^41^.

siXRCC4; AUAUGUUGGUGAACUGAGA^40^.

siRAD51C; AAGAGAAUGUCUCACAAAU^41^.

### Cleavage of plasmid by acetaldehyde

Plasmid pBluescript SK + was digested with EcoRV and precipitated using standard ethanol precipitation methods. The digested plasmid DNA was then resuspended in distilled water. Five hundred microliters of digested plasmid was incubated with the indicated concentrations of acetaldehyde at 37 °C for 3 h. Samples were separated on 0.7% agarose gels and stained with ethidium bromide.

### Preparation of persimmon fruit polyphenols and EGC

Persimmon fruit polyphenols were prepared as previously described^42^. Immature persimmon fruit cultivated in Japan was treated with ethanol to remove astringency. After homogenisation using a juice extractor, the homogenates were separated into four layers by centrifugation (1630×*g*, 15 min). The second layer from the bottom, containing tannin cells, was collected and lyophilised. The resulting powder was extracted with 100 volumes of water at 121 °C for 15 min and lyophilised again. Lyophilised polyphenols were dissolved in dimethyl sulfoxide (DMSO). Polyphenols from persimmon fruits used in this study consisted of 70% polyphenol (93% of which were proanthocyanidins) and 20% carbohydrate, as analysed using the Folin-Ciocalteu (using vanillin-HCl) and phenol-H_2_SO_4_ methods, respectively. Using a combination of thiolysis and liquid chromatography–mass spectrometry analysis^43^, the structure of persimmon polyphenols from the variety Tonewase, as major compounds of Kaki-tannin (Nara-type), have been reported to consist of EC, ECg, EGC, and EGCg at a ratio of 1:1:2:2 (50% galloylated)^44^. The catechins were polymerised with a B-type linkage, and their average molecular weight was calculated to be approximately 8000 Da, in a previous study^45^. ECg was determined to be the terminal unit. EGC (Wako, 059-08951) purified from green tea, was dissolved in DMSO and used at the indicated concentrations.

### Acetaldehyde concentration measurement

Acetaldehyde was incubated at 37 °C for 9 h in the presence or absence of EGC. An enzyme-linked acetaldehyde assay kit (BioAssay Systems, EFAC-100) was used to detect acetaldehyde in samples. Briefly, diluted samples were mixed with assay buffer, probe, NAD, and enzymes. After 30 min of incubation, the samples were analysed using an Infinite F200 Pro (TECAN, Switzerland) instrument.

### Statistical analysis

GraphPad Prism 7 was used for statistical analyses. For immunofluorescence, metaphase spread, and acetaldehyde detection experiments, unpaired *t* test, Mann–Whitney *U* test, and Tukey’s multiple comparison tests were used, respectively.

### List of chemicals/reagents

Acetaldehyde (Sigma(USA), 402788); Camptothecin (Wako(JAPAN), 038-18191); Formaldehyde (Sigma(USA), F1635); Epigallocatechin (Wako(JAPAN), 059-08951); KaryoMAX Colcemid Solution (Gibco(USA), 15212012); Antibiotic–Antimycotic Mixed Stock Solution (Nacalai(JAPAN), 02892-54); Opti-MEM (Gibco(USA), 31985062); PhosSTOP (Roche(Switzerland), 4906845001); Giemsa solution (Merck Millipore(USA), 1.09204.0503); Lipofectamine RNAiMAX Transfection Reagent (Invitrogen(USA), 13778100); Vectashield Mounting Medium (Vector Laboratories(USA), H-1000); BCIP-NBT Solution Kit for Alkaline Phosphatase Stain (Nacalai(JAPAN), 03937-60); EnzyFluo Acetaldehyde Assay kit (BioAssay Systems(USA), EFAC-100); Qubit Protein Assay Kits (Invitrogen(USA), Q33211); Qubit 1 × dsDNA HS Assay Kit (Invitrogen(USA), Q33230); Anti-CHK1-pS345 antibody (Cell Signaling (USA), 2348); Anti-Tubulin antibody (Sigma (USA), T4026) ; Anti-γH2AX antibody (Merck Millipore (USA), 05-636); Anti-RAD51 antibody (Merck Millipore (USA), ABE257); Anti-53BP1 antibody (Novus Biologicals (USA), NB100-304); Anti-RAD51C antibody (Novus Biologicals (USA), NB100-177); Alexa Fluor 594 goat anti-mouse (Invitrogen (USA), A11032); Alexa Fluor 488 goat anti-rabbit (Invitrogen (USA), A11034); Anti-Mouse IgG (H + L), AP conjugated (Promega (USA), S3728); Anti-Rabbit IgG (H + L), AP conjugated (Promega (USA), S3738).

## Supplementary Information

Supplementary Figures.

## Data Availability

The datasets generated in the current study are available from the corresponding author upon reasonable request.

## Abbreviations

ADH1B: Alcohol dehydrogenase 1B
ALDH2: Aldehyde dehydrogenase 2
ARK assay: Advanced recovery of K [potassium]-SDS precipitates assay
BRCA1: Breast cancer type 1 susceptibility protein
BRCA2: Breast cancer type 2 susceptibility protein
CHK1: Cell cycle checkpoint kinase 1
DPC: DNA–protein cross-link
DSB: DNA double-strand break
EGC: Epigallocatechin
ERCC1: Excision repair cross-complementation group 1
FA: Fanconi anemia
FANCD2: Fanconi anemia complementation group D2
γH2AX: Phosphorylated histone H2AX
HDR: Homology-directed repair
HR: Homologous recombination
G4: G-quadruplex
ICL: Interstrand crosslink
Ku70/80: 70-KDa and 80-kDa subunits of Ku protein
LIG4: DNA ligase 4
MRE11: Meiotic recombination 11
NBS1: Nijmegen breakage syndrome 1
NHEJ: Non-homologous end-joining
PARP: Poly(ADP-ribose) polymerase 1
RADAR assay: Rapid DNA adduct recovery assay
RAD51: RADiation sensitive 51
RAD51C: RAD51 paralog C
RIF1: RAP1 interacting factor homolog
ssDNA: Single-stranded DNA
XPF: Xeroderma pigmentosum, complementation group F
XRCC4: X-ray repair cross complementing 4
53BP1: Tumor protein p53 binding protein 1
