# Supplementary material for: Polyphenols from persimmon fruit attenuate acetaldehyde-induced DNA double-strand breaks by scavenging acetaldehyde
Source: Sci Rep. 2022 Jun 18;12:10300. doi: 10.1038/s41598-022-14374-9 (PMC9206672; doi:10.1038/s41598-022-14374-9)
Supplement: Supplementary file 1 — Supplementary Figures. [file 41598_2022_14374_MOESM1_ESM.docx]

**Supplementary Figures**

**Fig. S1**


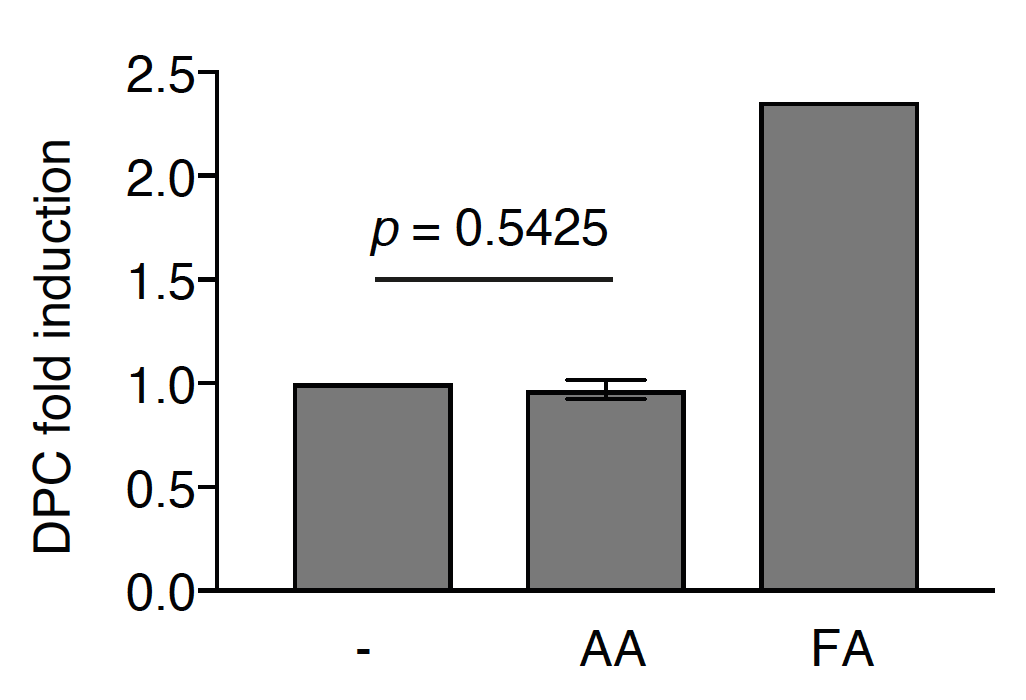


**Supplementary Fig. 1 Quantification of acetaldehyde-induced DPCs (RADAR assay).**

Quantification of DPCs derived from cells treated with the indicated concentrations of aldehydes. DPC amounts were analysed using RADAR assays. DPC -fold induction was calculated by normalizing the DPC amount of each sample against that of untreated samples. Statistical significance was determined using Mann-Whitney *U*-test. Error bars indicate SD (n = 3).

**Fig. S2**


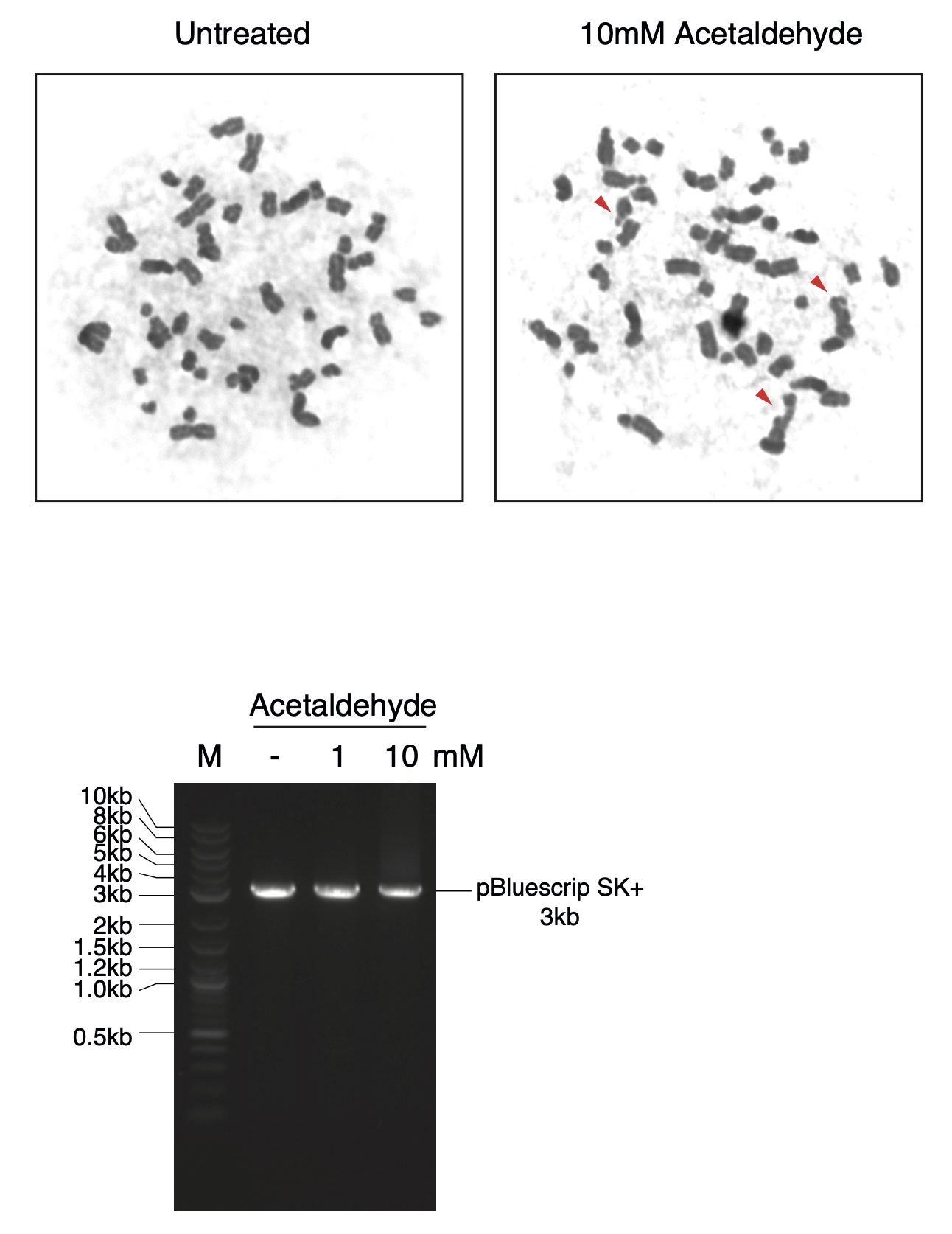


**a**

**b**

**Supplementary Fig. 2 Chromosome breaks and DNA cleavage induced by acetaldehyde treatment.**

**a** Representative images of metaphase chromosomes with breaks in the presence or absence of 10 mM acetaldehyde. Arrow indicates metaphase with chromatid/chromosome breaks. Quantification is shown in Fig. 2f.

**b** Plasmid cleavage by acetaldehyde. Linearised pBluescript SK+ plasmid was incubated with acetaldehyde and analysed using agarose gel electrophoresis. Gels were stained with ethidium bromide. “M” indicates molecular weight marker.

**Fig. S3**

**Supplementary Fig. 3 Uncropped scans for Western blots and agarose gel.**
